# A French multicentric prospective prognostic cohort with epidemiological, clinical, biological and treatment information to improve knowledge on lymphoma patients: study protocol of the “REal world dAta in LYmphoma and survival in adults” (REALYSA) cohort

**DOI:** 10.1186/s12889-021-10433-4

**Published:** 2021-03-02

**Authors:** Hervé Ghesquières, Cédric Rossi, Fanny Cherblanc, Sandra Le Guyader-Peyrou, Fontanet Bijou, Pierre Sujobert, Pascale Fabbro-Peray, Adeline Bernier, Aurélien Belot, Loic Chartier, Luc-Matthieu Fornecker, Isabelle Baldi, Krimo Bouabdallah, Camille Laurent, Lucie Oberic, Nadine Morineau, Steven Le Gouill, Franck Morschhauser, Corinne Haioun, Gandhi Damaj, Stéphanie Guidez, Gaëlle Labouré, Olivier Fitoussi, Laure Lebras, Rémy Gressin, Gilles Salles, Loïc Ysebaert, Alain Monnereau

**Affiliations:** 1 Hospices Civils de Lyon, Lyon Sud Hospital, 165 Chemin du Grand Revoyet, 69310 Pierre-Bénite, France; 2 CHU Dijon, 10 Boulevard Maréchal De Lattre De Tassigny, 21000 Dijon, France; 3 LYSARC, 165 Chemin du Grand Revoyet, 69310 Pierre-Bénite, France; 4 Inserm U1219 - EPICENE team, Université de Bordeaux, Bordeaux, France; 5 Bergonié Institute, 229 Cours de l’Argonne, 33076 Bordeaux, France; 6 CHU Nîmes, 4 Rue du Professeur Robert Debré, 30029 Nîmes, France; 7 Cancerology Institute Strasbourg Europe, Avenue Molière, BP 428, 67098 Strasbourg, France; 8 CHU Bordeaux, Avenue Magellan, 33604 Bordeaux, France; 9 Toulouse Research Center in Cancerology, 2 Avenue Hubert Curien, 31037 Toulouse, France; 10 IUCT Oncopole, 1 Avenue Irène Joliot Curie, 31100 Toulouse, France; 11 CHD Vendée, Boulevard Stéphane Moreau, 85000 La Roche-sur-Yon, France; 12 CHU Nantes, 1 place Alexis Ricordeau, 44093 Nantes, France; 13 CHRU Lille, Rue Michel Polonovski, 59037 Lille, France; 14 Henri Mondor Hospital, 51 Avenue du Maréchal de Lattre de Tassigny, 94010 Créteil, France; 15 Hematology Institute of Basse Normandie, 6 Avenue Côte de Nacre, 14033 Caen, France; 16 CHU Poitiers, 2 rue de la Milétrie, 86021 Poitiers, France; 17 CH Libourne, 112 Rue de la Marne, 33500 Libourne, France; 18 Polyclinique Bordeaux Nord Aquitaine, 15-35 Rue Claude Boucher, 33300 Bordeaux, France; 19 Léon Bérard Center, 28 rue Laennec, 69008 Lyon, France; 20 CHU Grenoble, Bd de la Chantourne BP 217, 38043 Grenoble, France

**Keywords:** Cohort study, Outcomes, Lymphoma patients, Real life, France

## Abstract

**Background:**

Age-adjusted lymphoma incidence rates continue to rise in France since the early 80’s, although rates have slowed since 2010 and vary across subtypes. Recent improvements in patient survival in major lymphoma subtypes at population level raise new questions about patient outcomes (i.e. quality of life, long-term sequelae). Epidemiological studies have investigated factors related to lymphoma risk, but few have addressed the extent to which socioeconomic status, social institutional context (i.e. healthcare system), social relationships, environmental context (exposures), individual behaviours (lifestyle) or genetic determinants influence lymphoma outcomes, especially in the general population. Moreover, the knowledge of the disease behaviour mainly obtained from clinical trials data is partly biased because of patient selection.

**Methods:**

The REALYSA (“REal world dAta in LYmphoma and Survival in Adults”) study is a real-life multicentric cohort set up in French areas covered by population-based cancer registries to study the prognostic value of epidemiological, clinical and biological factors with a prospective 9-year follow-up. We aim to include 6000 patients over 4 to 5 years. Adult patients without lymphoma history and newly diagnosed with one of the following 7 lymphoma subtypes (diffuse large B-cell, follicular, marginal zone, mantle cell, Burkitt, Hodgkin, mature T-cell) are invited to participate during a medical consultation with their hematologist. Exclusion criteria are: having already received anti-lymphoma treatment (except pre-phase) and having a documented HIV infection. Patients are treated according to the standard practice in their center. Clinical data, including treatment received, are extracted from patients’ medical records. Patients’ risk factors exposures and other epidemiological data are obtained at baseline by filling out a questionnaire during an interview led by a clinical research assistant. Biological samples are collected at baseline and during treatment. A virtual tumor biobank is constituted for baseline tumor samples. Follow-up data, both clinical and epidemiological, are collected every 6 months in the first 3 years and every year thereafter.

**Discussion:**

This cohort constitutes an innovative platform for clinical, biological, epidemiological and socio-economic research projects and provides an opportunity to improve knowledge on factors associated to outcome of lymphoma patients in real life.

**Trial registration:**

2018-A01332–53, ClinicalTrials.gov identifier: NCT03869619.

**Supplementary Information:**

The online version contains supplementary material available at 10.1186/s12889-021-10433-4.

## Background

Lymphomas comprise a heterogeneous group of more than 80 distinct entities classified on the basis of morphological, phenotypic, genotypic, and clinical characteristics [1]. Their age-adjusted incidence rates worldwide are more elevated in the most developed countries [2]. In France, they represent two thirds of hematopoietic cancers with an estimated 28,000 incident cases in 2018 (2000 Hodgkin lymphoma (HL) and 26,000 Non-hodgkin lymphoma (NHL)) [3]. The time trends in incidence over the last 30 years show an increase for most of the lymphoma subtypes (e.g. HL, follicular lymphoma (FL), diffuse large B-cell lymphoma (DLBCL), Marginal Zone Lymphoma (MZL) or Non-cutaneous mature T-cell lymphoma) [3]. This increase may be partly explained by the growing and ageing general population, as well as a better access to diagnosis and treatment. But other risk factors are suspected to be involved in the rising incidence [3]. Epidemiological studies have identified various factors associated with lymphoma onset, including socio-demographic factors, infectious disease status, family and medical history, lifestyle as well as occupational exposures (reviewed in Morton et al. [4]). There is a large etiologic heterogeneity among subtypes, with shared and distinct factors depending on subtypes, suggesting both subtype-specific and shared underlying mechanisms.

As for patient care, treatment efficacy greatly varies depending on histological lymphoma subtypes. Over the past 15 years, numerous therapeutic innovations have marked the treatment of lymphomas. For example, overall survival of patients with some main subtypes, including DLBCL, improved substantially in the last two decades with the introduction in 2003–2006 of anti-CD20 monoclonal antibodies in combination with chemotherapy as a first-line treatment [5, 6]. These innovations impacted the survival of patients with lymphoma, estimated in the general population. In France, data from population-based cancer registries (PBCR; FRANCIM network) showed an improvement in net survival over time clearly observed for DLBCL and FL (+18% between 1995 and 2010) whereas Hodgkin lymphoma survival remained stable (although very favorable) [7]. These encouraging results concerning the survival of lymphoma were also observed at European level [8].

Despite considerable improvement of treatment efficacy, our knowledge of factors associated with response to treatment or survival is currently limited and mainly based on factors related to the disease or its impact on the patient. Indeed, several factors linked to patients’ prognosis (e.g. histological subtype, staging, extra-nodal involvement, tumor size, high level of serum lactate dehydrogenase) and with patients’ characteristics (e.g. age, performance status) are well known and integrated in routine clinical care [9, 10]. There are also major developments in the research of prognostic markers in relation with lymphoma pathogenesis, but there is currently no consensus regarding use of these biomarkers for therapeutic decisions in real-life settings [11, 12]. Several epidemiological factors (e.g. medical history, lifestyle including physical activity, family history, quality of life) have been explored for patients’ prognosis, but the results have been rather inconsistent so far [13–24]. Interestingly, a recent publication identified in a retrospective analysis an association between occupational exposure to pesticides and response to treatment among DLBCL patients [25], but these results would need to be replicated in larger population-based prospective studies. Finally, several genome-wide association studies (GWAS) identified genome wide significant constitutional single nucleotide polymorphisms (SNPs) at risk for lymphoma, but the role of host genetic background in relation to patient outcome was less studied [26, 27].

Consequently, few studies have recently addressed the extent to which factors like socioeconomic status, social institutional context (i.e. healthcare system), social relationships, occupational and domestic exposures, individual behaviours, lifestyle or genetic determinants are associated with response to lymphoma treatments and patients’ survival in the general population. Moreover, as the number of lymphoma survivors is increasing, it raises new questions at population level about survivorship, including long-term sequelae of treatments and quality of life.

From a methodological point of view, most of the knowledge on disease behavior and treatment efficacy comes from clinical trials. But because of stringent inclusion criteria, most lymphoma patients are not included in clinical trials, and patients above a certain age, with comorbidities or already receiving some medications are usually excluded. Consequently, the generalizability of findings to the global lymphoma patient population is limited [28]. Thus, to complement these, researchers and stakeholders are now interested in evidence from real-world data (RWD) [29], i.e. data generated during routine clinical practice obtained outside the context of randomized clinical trials (RCT) [30]. For lymphoma patients, RWD may come from a variety of sources such as cancer registries or institutional databases, but these sources often provide limited access to clinical information, often no information on routine care, no epidemiological data, no biological samples or no follow-up data over years after the diagnosis.

In this context, the “REal world dAta in LYmphoma and Survival in Adults” (REALYSA) study was initiated in 2018 to fill this gap. The general objective of this study is to investigate in real life the prognostic value of epidemiological, clinical and biological factors for patients with lymphomas in France. Various indicators (survival, progression-free survival, treatment response rates and treatment-related toxicities, second cancer and appearance of new comorbidities) and patient reported outcomes (PRO) (e.g. quality of life (QoL), social support) will be estimated. The prognostic impact of clinical and epidemiological exposure factors at baseline on various outcomes will be measured. Access to healthcare and health behavior (e.g. screening, care consumption, type of follow-up and medical exams) will be described. The details of all treatment lines received will be documented. This study also has complementary biological objectives with the global aim to foster national and international research projects in order to identify new prognostic factors: (i) to create a virtual tumor library; (ii) to establish a centralized biological collection of peripheral blood.

## Methods and design

### Study design

**Fig. 1:**
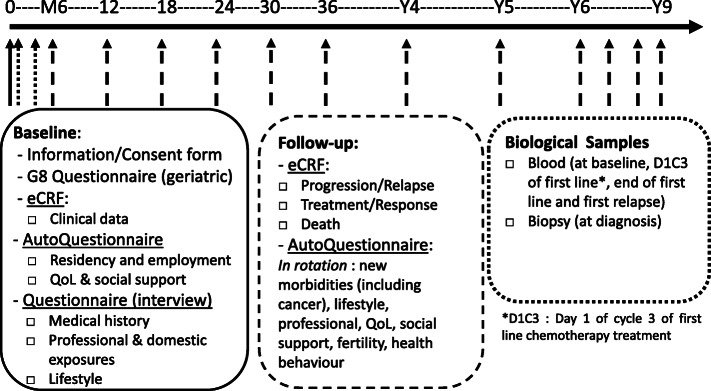
Study design of REALYSA

REALYSA is a real-life observational multicentric cohort (registered in the French Jardé Law as a research involving the human person of category 2 (RIPH2): interventional research involving only minimal risks and constraints). We aim to recruit 6000 patients over 4 to 5 years. The recruitment started in November 2018. The duration of the study is 9 years (4 to 5 years of recruitment (2018–2023) and between 4 and 9 years of follow-up, depending on the date of patient recruitment). The expected end of study is December 2028. Study design is described in Fig. 1. Patients are treated according to standard of care and no additional examination is required for the study, except for blood samples for subsequent biological analyses.

### Recruiting centers

**Fig. 2:**
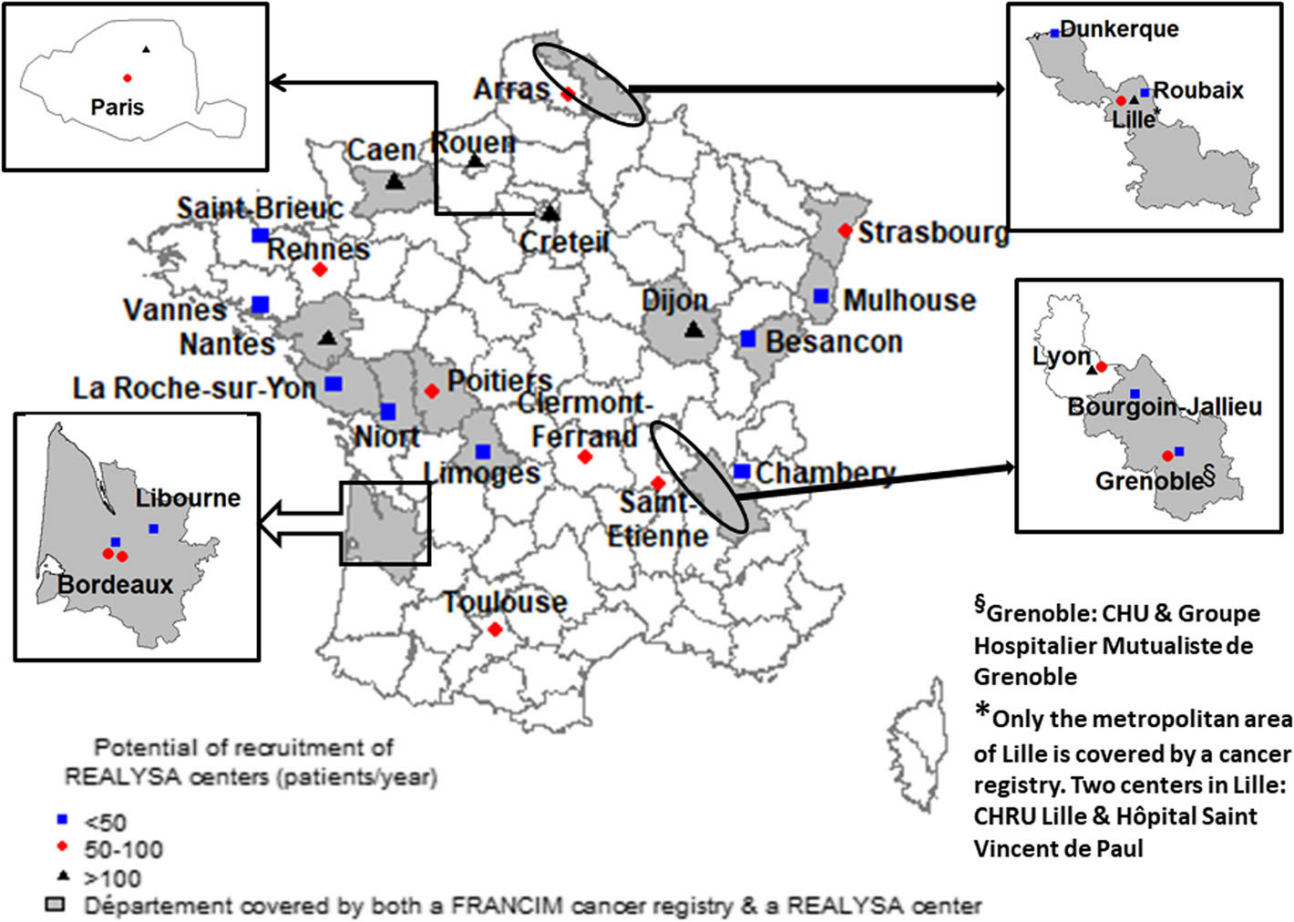
Recruiting centers in REALYSA study. Complementary information: The figure has been created by our own team with open source R software (version 4.0.2), using the packages maptools, raster, maps and mapdata

The recruiting centers meet at least one of these two criteria (see Fig. 2): 1) being an active center of the Lymphoma Study Association (LYSA) network with a good potential of patient recruitment and robust research facilities; 2) being located in a geographical area (i.e. French *département*) covered by a population-based cancer registry (PBCR). LYSA is the French cooperative group on lymphoma, federating researchers and medical practitioners from more than 80 healthcare centers throughout the country, in order to promote clinical research on lymphoma as well as improve prevention, management and treatment of lymphoma patients. Regarding registries, the French cancer surveillance system is an opt-out system, so any patient with a confirmed diagnosis of cancer and living in areas covered by a population-based cancer registry is automatically registered without specific patient consent, thus ensuring the exhaustivity of cancer incidence registration in these areas. PBCR are organized in a collaborative network named FRANCIM [31]. The main objectives of this network are to coordinate the 14 general cancer registries and 11 specialized cancer registries, to harmonize patient’s registration and data quality, to provide epidemiological indicators (incidence, survival, prevalence) and to coordinate epidemiological surveillance and research on cancer. PBCR will be useful to assess and eventually improve representativeness of the REALYSA cohort. Thirty-five French hospitals/clinics are currently participating to the study, including 18 (51%) large University Hospitals, 10 (29%) smaller general hospitals, 4 (11%) cancer centers and 3 (9%) private healthcare facilities. Other centers may open during the recruitment phase, if deemed necessary.

### Pilot and extension phase

In order to assess the feasibility of the project in a real-life setting (i.e. standard hospital service), a pilot phase was conducted in seven initial centers between November 2018 and June 2019. More than 300 patients were recruited during this timeframe. An evaluation of the pilot phase was conducted based on this recruitment [32]. An average of 47 patients per month had been recruited and data at baseline proved to be of high quality (e.g. 84% completion on average). The biological samples had been collected for over 80% of the included population. The histological distribution was found to reasonably match the national one [3]. Similar results were found for the median age and age distribution within the various histological subtypes. Regarding the epidemiological questionnaires, over 85% of the quality of life and social support data had been collected and approximately 50% of the interview had been performed (a 3-month delay is given to the centers to perform the interview, explaining this lower percentage at the time of the cut-off). In addition to this quantitative approach, a qualitative evaluation was performed through an online survey and phone calls with Clinical Research Assistants (CRA) in charge of the study in the recruiting centers. It showed a satisfactory integration of the program in routine care, a good compliance with study guidelines and no major difficulty regarding patient recruitment, data access or entry, as well as biological sample management. This analysis proved that the program was operational for an extension phase with minor modifications. For instance, the option to report continuous treatment (per os) was added in the eCRF, and a few items were simplified in the questionnaire to streamline the interview. Visual tools were also prepared to help the patient fill out the questionnaire. These adjustments were approved by the ethics committee at the end of 2019.

The extension phase was then launched in December 2019, with the opening of nine additional centers. A third phase of opening started in November 2020, with the opening of nineteen additional centers, including smaller hospitals.

### Study population

#### Inclusion criteria

Patients meeting all the following criteria are considered for enrollment in the study:

- Signature of the REALYSA consent form;
- Aged over 18 at the time of inclusion;
- Newly diagnosed with lymphoma in the last 6 months (180 days);
- Lymphoma subtype belonging to at least one of the 7 histological subtypes: Diffuse large B-cell lymphoma, Follicular lymphoma, Mantle cell lymphoma, Marginal zone lymphoma, T-cell lymphoma, Hodgkin’s lymphoma, Burkitt lymphoma.

#### Exclusion criteria

Patients meeting any of the following criteria are excluded from enrollment:

- Anti-lymphoma treatment already received (except pre-phase therapy: typically, corticosteroids, vincristine, cyclophosphamide and rituximab, alone or in combination);
- Documented HIV infection.

Details regarding included and excluded lymphoma subtypes can be found in Additional file 1.

### Inclusion procedure

Eligible patients are invited to participate in the study during a medical consultation with their hematologist. Patients are given detailed information regarding the project, including the follow-up modalities. If they agree to participate, they sign an informed consent form. The investigators then register the patient directly on the data capture system through the internet network.

In parallel, the pathological report of each patient included in REALYSA is sent to the coordinating center of the French cancer registries at Bordeaux (see Additional file 2). These data will be compared with incident cases collected by registries to analyze the representativeness of the population included in the REALYSA program.

### Data collection and management

#### Baseline

Collected data at baseline include clinical data on patient medical history and lymphoma diagnosis, lifelong history of residences and occupations (these self-administered questionnaires will gather the complete occupational history for each job held for at least 6 months as well as residential history for each place occupied for at least 1 year), as well as professional and domestic exposures, leisure time activities, lifestyle factors and women’s health (epidemiological questionnaire during a face-to-face interview) (see Table 1). Moreover, self-administered questionnaires assessing QoL and social support are filled in by the participants. Finally, the G8 questionnaire (i.e. geriatric patient screening test for elderly patients with cancer [33]) is administered by the investigators to patients over 70 years old. Clinical and treatment data are extracted from the patient medical record and entered in an electronic case report form (eCRF) through a secure web-based platform (Clinical Data Management System Ennov®) by the CRA in the participating centers. Data regarding quality of life, social support and G8 questionnaire are also entered directly in the eCRF. Epidemiological and other self-administered questionnaires are sent to the University of Bordeaux for centralized data entry in a dedicated epidemiological database (Redcap®, 9.5.6 Vanderbilt University) to ensure data homogeneity.

**Table 1:** Overview of collected data at baseline

Collected data	Data source	Data entry system	Staff member for data entry
Demographics	Patient medical record	eCRF	CRA^a^
Care pathway	Patient medical record	eCRF	CRA
Medical history and concomitant treatments	Patient medical record	eCRF	CRA
Characteristics at initial diagnosis^b^	Patient medical record	eCRF	CRA
Lifelong history of residences	Auto-questionnaire	Epidemiological database	Data entry operator
Lifelong history of occupations	Auto-questionnaire	Epidemiological database	Data entry operator
Medical History^c^	Epidemiological questionnaire (interview)	Epidemiological database	Data entry operator
Professional and domestic exposures	Epidemiological questionnaire (interview)	Epidemiological database	Data entry operator
Lifestyle	Epidemiological questionnaire (interview)	Epidemiological database	Data entry operator
Women health	Epidemiological questionnaire (interview)	Epidemiological database	Data entry operator
Quality of life	Auto-questionnaire (QLQ-C30 + lymphoma specific modules)	eCRF	CRA
Social support	Auto-questionnaire (SSQ6)	eCRF	CRA
Geriatric screening	Screening questionnaire (G8) performed by the investigator	eCRF	CRA

^a^Clinical Research Assistant

^b^including date, clinical/biological details of the pathological diagnosis, nodal/extra-nodal involvement, exams performed, staging, hematology and biochemistry laboratory data, serologies

^c^including personal history of infectious diseases, allergies, cancers, chronic diseases, treatments, imaging and family history of hematological malignancies

#### Follow-up

Timepoints for data collection during follow-up are described in Table 2. Clinical data, as well as lifestyle changes, new morbidities, professional situation and work stress, infertility issues, use of alternative medicine are collected using data from medical record or via self-administered questionnaires filled in by the patients. Follow-up of patients is performed every 6 months during the first 3 years and annually thereafter until year 9. Due dates for completing the follow-up are generated from the diagnosis date (i.e. date of biopsy).

**Table 2:** Overview of data collection timepoints during follow-up

Collected data	Data source	M6	M12	M18	M24	M30	M36	Y4	Y5	Y6	Y7	Y8	Y9
Disease status at last hematology consultation	Patient medical record	x	x	x	x	x	x	x	x	x	x	x	x
Number of consultations and imaging exams	Patient medical record	x	x	x	x	x	x	x	x	x	x	x	x
Treatment lines^a^	Patient medical record	x	x	x	x	x	x	x	x	x	x	x	x
Response to treatment	Patient medical record	x	x	x	x	x	x	x	x	x	x	x	x
Adverse events	Patient medical record	x	x	x	x	x	x	x	x	x	x	x	x
Relapses/progressions/transformations^b^	Patient medical record	x	x	x	x	x	x	x	x	x	x	x	x
New malignancy	Patient medical record	x	x	x	x	x	x	x	x	x	x	x	x
New morbidities	Auto-questionnaire	x		x		x		x	x	x	x	x	x
Lifestyle	Auto-questionnaire	x		x				x		x		x	
Professional situation	Auto-questionnaire		x		x				x		x		x
Work stress	Auto-questionnaire (Siegrist questionnaire)		x		x				x		x		x
Quality of life	Auto-questionnaire (QLQ-C30 + lymphoma specific modules)		x		x		x			x			x
Social support	Auto-questionnaire (SSQ6)	x	x		x		x	x	x	x	x	x	x
Women sexual and reproductive health	Auto-questionnaire					x							
Health behaviors^c^	Auto-questionnaire						x			x			x
Date and cause of death	Patient medical record												
End of study: reasons for early termination	Patient medical record												

^a^detailed therapies, start/end dates, dose modality (full/reduced in chemotherapy), amount of Gray if radiotherapy, transplant and other surgeries

^b^dates, involvement, available documentation, method of evaluation used, staging

^c^including use of alternative medicine, screening behaviors

Questionnaires are either mailed by the CRA or given to the patient during a medical consultation for regular follow-up. Patients will give back to the CRA the questionnaires or mail them to the center using a pre-paid envelope. If questionnaires are not returned by patients in a timely manner, CRA and their hematologist will contact the patient by phone to motivate him/her to fill in and return the questionnaires.

CRA are in charge of follow-up data entry into the eCRF.

#### Measurement instruments

Several measurement instruments are used in this program.

Quality of Life is assessed using the EORTC (European Organisation for Research and Treatment of Cancer) QLQ-C30 questionnaire together with three lymphoma-specific modules [34]:

- For patients with Hodgkin Lymphoma: QLQ-HL27;
- For patients with Non-Hodgkin Lymphoma - High Grade: QLQ-NHL-HG29;
- For patients with Non-Hodgkin Lymphoma - Low Grade: QLQ-NHL-LG20.

Five dimensions may be assessed: (i) symptom burden due to disease and/or treatment; (ii) neuropathy (only for NHL-High Grade); (iii) symptomatic scale for physical fatigue; (iv) emotional impacts; and (v) worries/fears about health and functioning.

Social support is measured using the French validated version of the SSQ6 questionnaire [35, 36]. This questionnaire measures two dimensions of perceived social support: (i) the availability of social support; and (ii) the satisfaction regarding this support.

The G8 geriatric screening tool is used by the investigator during the medical consultation to identify elderly patients (> 70 years) who could benefit from comprehensive geriatric assessment [33].

Work stress will be estimated using the Siegrist questionnaire (short version) [37].

#### Pathology review

We collect date of biopsy, the methods of biopsy (e.g. excision, core) and the initial pathological report. The diagnosis of enrolled lymphoma patients is based on the pathology report and are classified according to World Health Organization (WHO) criteria [1]. Because a national pathology review by a panel of hematopathologists is already organized in France for lymphoma within the Lymphopath histopathological network, we will cross-check each new diagnosis with this network of experts that validate the diagnosis of more than 70% of French lymphoma cases [38]. For patients also included in LYSA clinical trials, the centralized diagnosis review performed for the trial will be cross-checked with the REALYSA data. In addition, for specific studies on REALYSA database, some extracted cases could be reviewed by expert hematopathologists.

### Project coordination

The project is sponsored by the Hospices Civils de Lyon (HCL) and coordinated by the LYSA and its academic clinical research association LYSARC based in Lyon, together with the Inserm unit EPICENE (public research unit specialized in cancer epidemiology and environmental exposures) based in Bordeaux. There are two principal investigators (one professor of hematology based in Hôpital Lyon Sud and one expert in lymphoma epidemiology based in the Gironde registry as well as EPICENE team in Bordeaux), and each principal investigator is responsible for leading its study component (i.e. clinical component and epidemiological component), working in close collaboration with the study team.

The governance is shared between two committees. The Scientific Committee is a working group of academic members chosen by LYSA/RC and EPICENE for their scientific expertise in epidemiology, statistics, clinical medicine or biology. Its main mission is to ensure the consistency and scientific quality of REALYSA. It is also in charge of evaluating the relevance and scientific quality of projects using REALYSA data. Moreover, all communications and scientific publications of REALYSA and of projects using REALYSA data are reviewed by the Scientific Committee. The Steering Committee is composed of members of the LYSA/RC and EPICENE, representative of the HCL, representatives of the investigating centers (principal investigator and representatives of DRCI (Department of Clinical Research and Innovation, i.e. department managing clinical research projects in hospitals)) and representatives of industrial companies supporting the implementation of REALYSA. Its main mission is to discuss the progress of the project implementation and the areas for improvements.

The routine coordination is ensured by the study team, composed of the two coordinating investigators, three project managers, a biostatistician and two data managers. The study team is in charge of producing and providing the study documents, ensuring the regulatory compliance, assisting the recruiting centers, as well as ensuring data completion and quality. It also coordinates the submission process of projects using REALYSA data, including the review by the Scientific Committee.

For each project aiming at analyzing REALYSA data or samples, there will be a project leader with relevant expertise. This project leader will submit a project to the REALYSA Scientific Committee, and if the project is validated, the project leader will work with the study team to implement the project.

### Data quality

#### Data completion and homogeneity

To ensure the best level of data completion and homogeneity, a number of tools have been developed by the study team and shared with the centers:

- detailed completion guidelines for clinical eCRF and epidemiological questionnaires;
- Standard Operating Procedures for patient inclusion, template of excel sheets to plan follow-up timepoints, visuals for face-to-face interviews (e.g. contraceptive packaging);
- regular contacts between the project managers and the CRA: in-person initiation visit (4 h), check-up calls after the first interviews, CRA meetings (on the sidelines of the LYSA congress);
- regularly updated Frequently Asked Questions (FAQ);
- listings of missing data or missing documents and upcoming follow-up timepoints.

Moreover, a center specific report summarizing the data of the centers and comparing their data to the whole cohort population is produced and sent to each center twice a year. Finally, local investigators are all part of the Scientific Committee and receive the minutes.

#### Data validation

A strong data validation system inspired from clinical research standards has been implemented and is regularly running, with different levels of data checking. First, the electronic data entry system contains automatic checks performed at regular intervals for data completeness and consistency. Second, a scientific review is conducted by the study team to guarantee scientific coherence of data. Last, a medical review is performed by center investigators to ensure overall clinical data coherence of major endpoints (e.g. treatment plan, staging, response to treatment, events during the follow-up like progression, relapse or death). In case of incoherence and when deemed necessary, queries are generated and sent to the centers. Corrections are edited in the eCRF and tracked in the audit trail. At regular intervals, the overall completeness and quality of the data is assessed.

Epidemiological data are systematically checked for aberrant or missing data. If there is extensive missing data or if clarification is needed, the CRA in charge of the interview is contacted.

### Biological samples

For the first 2500 patients included in REALYSA, blood samples will be collected at baseline (before any treatment), during first-line treatment, at end of first-line treatment and at relapse. Additional blood samples are also collected for patients with anaplastic lymphoma kinase positive (ALK+) anaplastic large cell lymphoma (ALCL), for specific analyses on antibodies and nucleophosmin (NPM) transcript. Details are presented in Table 3. In case of premature first line treatment discontinuation (before cycle 3), the samples will be drawn at the time of treatment discontinuation.

**Table 3:** Biological sampling plan

Sampling	Banking constitutional DNA	Banking plasma	Banking serum	NPM-ALK^b^ (ALK+ ALCL)^c^	Anti-ALK Ab^d^ (ALK+ ALCL)
Baseline^a^	x	x	x	x	x
Day 1 Cycle 3		x		x	
End of first-line treatment		x		x	x
First relapse		x		x	

^a^before any treatment

^b^*NPM* Nucleophosmine

^c^*ALK+ ALCL* anaplastic lymphoma kinase positive anaplastic large cell lymphoma

^d^*Ab* Antibodies

### Virtual tumor biobank

The standard management of patients for their pathology includes taking tumor biopsies to establish the diagnosis. The remaining tumoral material (biopsy included in paraffin or frozen) will be requalified for research and stored by the centers. This material could be requested for specific projects in the future.

### Statistical analysis

#### Sample size calculation

No sample size calculation is strictly required for cohort studies but was needed for planning and funding perspectives. We have therefore used a pragmatic approach, based on one of the objectives of the study, which is to be able to detect an association between the exposure of interest and the clinical outcome of interest (e.g. response to treatment, progression-free survival, overall survival – see section on clinical outcomes) for a given lymphoma subtype. First, using lymphoma incidence rates [3, 39] in the geographical study zone, we estimated the number of new lymphoma cases that could be recruited in REALYSA over a 4-year recruitment period. Second, we calculated the hazards ratio (HR) that this study would be able to detect as a function of the number of events, which can be then back transformed to a number of patients of a given lymphoma subtype.

Firstly, based on the initially planned recruiting centers, we would expect 2796 new lymphoma cases yearly, so a total of 11,183 new cases during the 4 years of inclusion (unpublished data coming from an extraction of registry data). Considering a participation rate of 70% and an improving dynamic recruitment rate (40% during the 1st year, 75% the 2nd year, and 100% on the last 2 years), the number of cases that could be recruited in REALYSA is around 6000 patients (exactly 6165 patients, distributed as follows: 911 patients with HL, 2123 patients with DLBCL, 1294 patients with follicular lymphoma (FL), 344 patients with mantle cell lymphoma (MCL), 88 patients with Burkitt lymphoma, 958 patients with MZL and 447 patients with NHL-T).

Secondly, we calculated the detectable HR assuming a binary exposure of interest (say “present” vs “absent”). We relied on the proportional hazard model to describe the association between the exposure and the mortality hazard, with a 2-sided test at α = 0.05 (type 1 error rate) and a desired 80% power (i.e. 1 minus the type 2 error rate). With a pre-specified value for the prevalence of the exposure in our sample, we can obtain the detectable HR as a function of the number of events [40]. In order to get an absolute measure of the difference between groups (as opposed to the HR, which is a relative measure), one could use the link between HR and survival. This would allow to express the difference between the survival of the exposed, *S*_1_(*t*), and the unexposed *S*_0_(*t*) as: *S*_1_(*t*) = *S*_0_(*t*)^*HR*^.

**Fig. 3:**
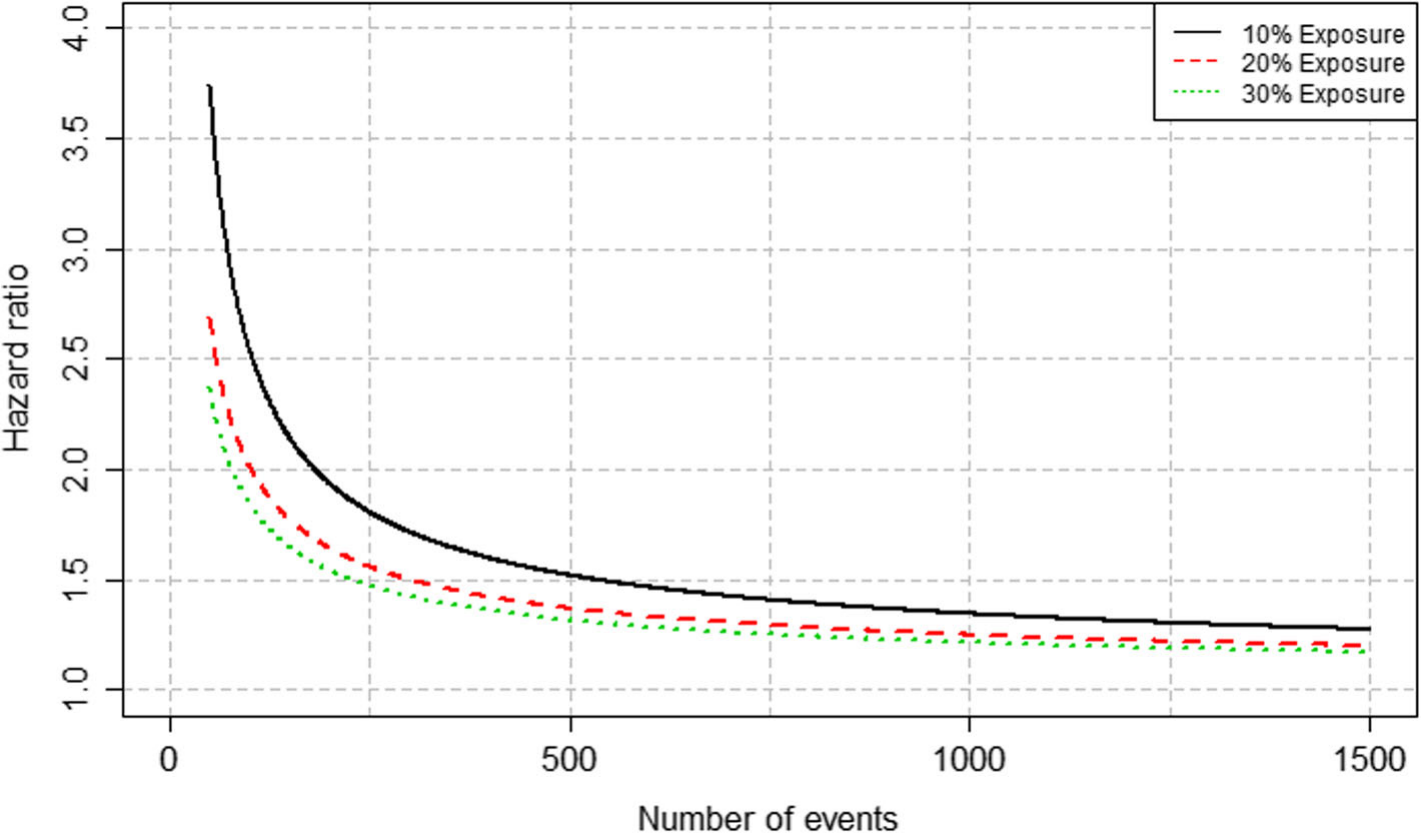
Detectable hazard ratio according to the number of events for different levels of exposure prevalence

We investigated scenarii with 3 levels of prevalence for the exposure: 10, 20 and 30%. The results are shown in Fig. 3 with the detectable HR according to the number of events. We would need to observe 530 events to be able to detect a HR of 1.5 with a type I error rate of 0.05, a power of 0.8 and an exposure prevalence of 10%. Therefore, if we assume that the proportion of events among the cases is approximately 30% (as observed for example for DLBCL for 1-year survival in France [7], and assuming no lost-to-follow up), then we need to observe 1767 patients with DLBCL (530/0.3). This HR of 1.5 in the context of DLBCL would then correspond to a 1-year survival of 70% in the unexposed group vs 59% in the exposed group. Despite the fact that this approach for sample size calculation relies on many assumptions, it has the advantage of being general and versatile to our different settings (i.e. according to lymphoma subtype and clinical outome of interest).

Consequently, the sample size was set at 6000 patients, as a good balance between feasibility and statistical power, at least for most common subtypes (i.e. DLBCL, FL and HL).

#### Representativeness analysis

One of the objectives of this program is to have a satisfactory representativeness of the included population as compared to patients diagnosed with lymphoma in the general population, thus allowing generalization of the results. Twice a year and for each sex-lymphoma subtype combination, we compare age distribution of incident cases between the national incidence estimates based on FRANCIM data [3] and the REALYSA cohort to monitor the evolution of representativeness. We also compare the distribution of the lymphoma subtypes between the national estimates and REALYSA. Comparison of the main confounders’ distributions (i.e. sex, age, lymphoma subtype, stage, performance status) is also done bi-annually between REALYSA centers, in order to identify potential bias in patients’ recruitment in specific centers. Moreover, two pilot *départements* (Gironde and Isère) covered by cancer registries have been chosen to cover an increased proportion of the population by opening 3 to 4 centers in the *département*. Specific analyses will be performed in these *départements* to conduct a fine-tuned analysis of the representativeness, which will be very helpful to adjust for the global cohort if necessary, using particular statistical techniques [41].

#### Clinical outcomes

The following clinical measures will be recorded at different timepoints: response to treatment, progression-free survival (PFS), event-free survival (EFS), time to next anti-lymphoma treatment (TTNLT), overall survival (OS). Other quantities of interest, such as the net survival, survival after progression, transformations, onset of second cancers will also be studied.

#### Generalities on the statistical methods for basic description of the association between the variables and time-to-event outcomes

Continuous variables will be summarized in tables displaying sample size, mean, standard deviation, median, and range; quartiles will also be presented when considered relevant. Categorical variables will be described in counts and percentages, including a specific category for missing data. Time to events will be described using Kaplan-Meier method, and survival probabilities (with the corresponding curves) will be provided with their 95% Confidence Interval (CI). For (semi-) competing risks setting (i.e. dealing with multiple event types), cumulative incidence functions will be our measure of interest.

Association between a categorical baseline prognostic factor and the time to event will be assessed by a two-sided log-rank test and quantified with a hazard ratio (HR) with 95% CI as estimated from a Cox model including only this variable as predictor. The continuous baseline prognostic factors will be categorized using the quantiles of their observed distribution and we will apply the same strategy as the categorical prognostic factors. Additionally, for the continuous factors, we will use their original version in a survival model (e.g. Cox or flexible parametric model) to estimate the (eventually time-dependent) HR associated with a 1-unit increase of the factor. Multivariable survival models will also be used for estimating conditional HRs for the main clinical and epidemiological factors. Time-updated prognostic factors will be analyzed using extensions of the survival models aforementioned.

Despite all the efforts made for representativeness, the cohort might end up not being representative of the French general population of lymphoma patients. In that case, statistical techniques such as covariate adjustment methods [42] or methods based on the Inverse probability weighting (IPW) technique [43] could be implemented to correct for this bias [41, 44]. In that regard, the REALYSA setting in which the recruiting centers are located in *départements* also covered by PBCR will be very useful.

#### Main prognostic epidemiological factors

For each topic, relevant indicators of exposure will be defined. As an example, the following topics will be analyzed to study their association with clinical outcomes (non-exhaustive list): tobacco smoking, alcohol consumption, socio-professional categories, medical history, medical family history of cancer, domestic exposures, occupational exposures. Additionally, for each analysis, potential confounding factors will be considered on a case-by-case basis, as they may influence the prognostic of lymphoma in the population, such as the international prognostic indexes, socio-economic status, lifestyle habits and treatments. Other potential confounding factors such as the population density, the presence of polluting industries, urban or rural status of the place of residence will be used whenever necessary. In case of a substantial amount of missing data in one or more potential confounders, we will rely on multiple imputation techniques.

### Ethics

The study is performed according to the declaration of Helsinki, and national laws and regulations for RIPH2 studies. The REALYSA study was approved by a French ethics committee (Comité de Protection des Personnes Ouest II - file number: 2018/46) and by the National Commission for data protection and freedom of information (CNIL - decision number: DR-2018-238). Written informed consent is obtained from patients before any data collection. A specific signed consent form is also obtained from each patient willing to participate in genetic studies that may be conducted on blood samples. Patients are free to refuse to participate or to withdraw from the study at any time. Collected data are anonymous and secure data management systems are used. Any substantial change of the protocol (e.g. number of centers, number of collected blood samples) will be validated by the ethics committee before implementation.

## Discussion

This cohort will include around 6000 patients with clinical, epidemiological and biological data. This initiative constitutes a great opportunity to set up and emulate collaborative research projects on a wide range of topics, with the overall aim of improving knowledge on lymphoma patients’ outcomes in real-life setting.

France is a particularly appropriate country to set up such study due to a global organization in diagnosis, clinical research and epidemiological surveillance of lymphoma patients. The LYSA is a very active and well-structured organization which brings together professionals specialized in the field of lymphoma across the country and plays a leading role in coordinating cutting-edge research projects on lymphoma. REALYSA also takes advantage of the presence of the national Lymphopath network to improve the quality of diagnosis of included patients [38]. As the LYSA is a group specialized in clinical trials in lymphoma, there is also a systematic diagnostic review for patients included in clinical trials by the hematopathologists of LYSA Pathology committee. For these reasons, we did not choose to centralize all tissue blocks as has been done in other lymphoma cohorts (e.g. LEO – SPORE [45]), but we created a virtual tumor bank for the accessibility of tumor tissues for specific studies. Finally, the close collaboration between REALYSA and FRANCIM offers an innovative opportunity for a formal evaluation of the representativeness of patients included in this prospective cohort by mapping data obtained from REALYSA inclusion and those recorded in registries. To the best of our knowledge, this type of prospective collaboration between clinical centers and registries is unique at a national level.

Other prospective cohorts are currently implemented such as the Haematological Malignancy Research Network (HMRN). In this population-based cohort, all hematological malignancies were prospectively included since 2004 in a specific geographical area of United Kingdom [46, 47]. The Lymphoma Specialized Program of Research Excellence (SPORE) Molecular Epidemiology Resource (MER) cohort enrolled prospectively patients from Mayo Clinic (Rochester, Minnesota) and the University of Iowa (Iowa City, Iowa) since 2002 [45]. Investigators extended this program in 2015 to eight US centers as part of the Lymphoma Epidemiology of Outcome (LEO) program (NCT02736357). The objectives of REALYSA cohort are close to the ones of these programs but we expect to have a cohort with as much as possible a national coverage with a control of the representativeness thanks to data from registries (see Fig. 2).

Although clinical trials remain the gold standard for the evaluation of new treatment options, a majority of lymphoma patients are currently treated outside clinical trials. In addition, large prospective phase III trials are now less frequent with the development of therapeutic trials targeting specific populations, for instance with a particular lymphoma pathogenesis. With comprehensive clinical/biological data collections, we will be able to determine the efficacy and toxicities of some treatment options performed in daily practice that avoid the problem of patient selection in clinical trials. For instance, we observed that the median age of DLBCL in general population is more than 70 years old [48], but was estimated near 60 years old in clinical trials or in clinic-based observational cohort from tertiary hospitals [49]. Comparison of patients with mantle cell lymphoma (MCL) included in clinical trials or registered in cancer registries showed that patients from clinical trials were younger and had less advanced stage; there was also an excessive mortality mainly in elderly MCL patients from registries confirming patient selection bias in clinical trials [50]. In this setting, we will also be able to prospectively validate clinical prognostic scores (e.g. FLIPI, MIPI) among real-life patients. The validation of clinical trial results in general population after approval is also a major challenge for new targeted therapies in lymphoma such as Chimeric Antigen Receptors (CAR) T-cells, new monoclonal antibodies or tyrosine kinase inhibitors regarding specificities of their uses, specific adverse effects and cost. For instance, in a recent study of the HMRN network, the impact of novel therapies in real-life settings for outcome of MCL patients was well documented [51]. In this context, a prospective cohort such as REALYSA will offer a better evaluation of new therapeutic options than retrospective studies with possibility of health-economic studies. For these new therapeutic options, a comparison between REALYSA patients included in clinical trials vs REALYSA patients not included in clinical trials will also be possible, as we collect information about clinical trial participation. The utility of RWD for the detection of rare or late toxicities is now well recognized [52]. For instance, after the publication of cardiac surveillance guidelines mostly based on RWD, the cardiac surveillance rate of lymphoma patients treated by anthracycline-based therapy seemed to improve [53]. The analysis of prospective RWD collections has informed clinical practice, in particular for rare lymphoma subtypes and for clinical situations for which designing clinical trials remains difficult: for instance, the modality of DLBCL and HL patient surveillance after first line therapy was modified by data coming from RWD showing the controversial use of routine CT-scan for the detection of relapses [54–56]; similarly, use of RWD contributed to the definition of new survival endpoints such as EFS24 in DLBCL or the comparison of patient life expectancy with general population [57–59].

Recently, there is a major effort to aggregate biological collections from several institutions or among consortium with samples coming from patients mostly treated in real-life setting. For instance, three major studies from a group of institutions investigated the relation between genome/exome sequencing with the prognosis of DLBCL patients [11, 60, 61]. In REALYSA, we will bank an important number of biological specimens, allowing ambitious biological and genetic studies to identify new biological markers from tumors but also from the host with germline DNA analyses. Interestingly, with the collection of clinical, biological and epidemiological data, we will be able to analyze the challenging question of the interaction between environmental exposure and tumor biology [62], as well as extend our previous works on the relation between inherent genetic variations analyzed by GWAS and prognosis [27].

The longitudinal evaluation of patients during follow-up is a major objective in our cohort. The collection of data regarding professional situation, reproductive health, health behaviors, appearance of new morbidities and evolution of QoL will be of great utility to describe and analyze specific challenges of daily living for lymphoma survivors in the general population. In this context, recent works in advanced-stage lung cancer showed that the self-reported symptoms during follow-up using a web-based application improved overall survival due to an early detection of relapse [63].

Several weaknesses may be identified in this study. First, unlike other cohorts (e.g. HMRN), not all lymphoid malignancies are included in REALYSA. We elect not to include patients with chronic lymphocytic leukemia (CLL)/lymphocytic lymphoma, primary central nervous system (CNS) lymphoma and post-transplanted lymphoma as there are other national networks for these lymphoid malignancies and their managements are very distinct from other NHL subtypes. Similarly, primary cutaneous T-cell lymphoma that are mainly diagnosed, treated and followed by dermatologists specialized in this disease are not included. Second, depending on the subtypes, the statistical power may be limited in some cases. Indeed, the comprehensive occupational and domestic exposure questionnaire, associated with clinical data, will help in understanding the role of environmental conditions (including socio-economic status, social institutional context, social relationships, environmental exposures, individual behaviors, lifestyle) on lymphoma prognosis. Previous studies suggested the potential prognostic impacts of some environmental exposures on lymphoma patient outcome [25, 62]. The major issue of these studies is to have sufficient statistical power to prove an association depending on exposure prevalence, the sample size of the lymphoma subtype and the number of events. We think that for the most frequent lymphoma subtypes, such as DLBCL, FL and HL, the power would be satisfactory to detect some environmental conditions as prognostic factors. We recognize that infrequent lymphoma subtypes or for low prevalence exposures, results will be considered as exploratory and will need further replications for instance through international collaborations.

Finally, there are three major challenges in this study. First, we will have to pay strong attention to recruitment dynamics and patient characteristics to avoid recruitment biases and to have a population as representative of the general population of lymphoma patients as possible. Strong guidelines are given to centers in order to recruit all patients meeting the inclusion criteria, without any distinction on age, general condition or clinical prognosis. Moreover, smaller and non-University centers were opened during the extension phase in order to capture a larger population of patients, including those treated outside University Hospitals. Interim analyses will be conducted in order to compare participants’ characteristics with registries data. Nevertheless, even if the cohort is not representative of the whole French population of lymphoma patients, we strongly believe that building such cohort will be of use for many research works despite the lack of representativeness, as long as the “scientific inference is still valid” [64, 65]. The second challenge is the retention of patients in the cohort. Prospective follow-up is tied to the diagnosis date, in order to follow clinical management (e.g. annual follow-up from diagnosis), thus facilitating patient engagement. Newsletters with information on the study and projects will be sent to patients, in order to create a sense of belonging to the study. A dedicated webpage has also been created and updated with information on study and projects. Lastly, we aim to involve patients as partners of this research in order to facilitate communication and feedbacks between the research team and the patients included in the cohort and eventually minimize the attrition rate. Finally, as for all real-world studies, data completeness remains a major challenge. However, this study being conducted by investigators and CRA belonging to the hematological departments, we trust that this will maximize our capacity to recover satisfactory data.

This cohort is a perfect framework for multidisciplinary projects, as well as national and international collaborations. The close partnership with the LEO study team [45] and InterLymph consortium [66] will facilitate international projects and comparative analysis. For some rare subtypes with low numbers (e.g. Burkitt lymphoma), data could be pooled with other international cohorts to obtain relevant and robust results for these rare lymphomas [67], as it was already and successfully performed in a large pooling initiative studying risk factors for 11 NHL subtypes on behalf of InterLymph consortium [68]. Proposals for collaborative research projects from all disciplines will be considered by the study team.

Trial status: recruiting.

Protocol version 2.0, date: 09-01-2020.

Start recruitment: 11-14-2018.

Approximate date recruitment completion: 11-14-2023.

## Supplementary Information

**Additional file 1.** Lymphoma subtypes included and excluded in the study, according to the 2016 WHO classification. Detailed list of lymphoma subtypes included and excluded of the study.

**Additional file 2.** Inclusion procedure in the REALYSA study. Overview of the inclusion procedure in the REALYSA study.

## Data Availability

The datasets generated during the current study are available from the corresponding author on reasonable request. As the REALYSA cohort is under a specific collaboration and data sharing plan, which encourages collaboration and use of the resource, external collaborators can contact the corresponding author. Whereas use is prioritized for researchers associated with the REALYSA cohort, all requests are considered by the Executive Committee.

## Abbreviations

CAR: Chimeric Antigen Receptor
CI: Confidence Interval
CLL: Chronic Lymphocytic Leukemia
CNIL: National Commission for data protection and Freedom of Information
CNS: Central Nervous System
CRA: Clinical Research Assistant
DLBCL: Diffuse Large B Cell Lymphoma
DNA: DeoxyriboNucleic Acid
eCRF: electronic Case Report Form
EFS: Event-Free Survival
EORTC: European Organisation for Research and Treatment of Cancer
FL: Follicular Lymphoma
GWAS: Genome Wide Association Study
HL: Hodgkin Lymphoma
HMRN: Haematological Malignancy Research Network
HR: Hazard Ratio
LEO: Lymphoma Epidemiology of Outcomes
LYSA: Lymphoma Study Association
NHL: Non-Hodgkin Lymphoma
OR: Odds Ratios
OS: Overall Survival
PBCR: Population-Based Cancer Registry
PBMC: Peripheral Blood Mononuclear Cell
PFS: Progression-Free Survival
PRO: Patient Reported Outcome
QoL: Quality of Life
RCT: Randomized Clinical Trial
REALYSA: REal world dAta in LYmphoma and Survival in Adults
SNP: Single Nucleotide Polymorphism
TTNLT: Time To Next anti-Lymphoma Treatment
WHO: World Health Organization
