# Supplementary material for: A French multicentric prospective prognostic cohort with epidemiological, clinical, biological and treatment information to improve knowledge on lymphoma patients: study protocol of the “REal world dAta in LYmphoma and survival in adults” (REALYSA) cohort
Source: BMC Public Health. 2021 Mar 2;21:432. doi: 10.1186/s12889-021-10433-4 (PMC7927409; doi:10.1186/s12889-021-10433-4)
Supplement: Supplementary file 1 — Additional file 1. Lymphoma subtypes included and excluded in the study, according to the 2016 WHO classification. Detailed list of lymphoma subtypes included and excluded of the study. [file 12889_2021_10433_MOESM1_ESM.docx]

***Lymphoma subtypes included in the study:***

Mature B-cell neoplasms

Splenic marginal zone lymphoma

Splenic B-cell lymphoma/leukemia, unclassifiable

Splenic diffuse red pulp small B-cell lymphoma

Extranodal marginal zone lymphoma of mucosa-associated lymphoid tissue (MALT lymphoma)

Nodal marginal zone lymphoma

Pediatric nodal marginal zone lymphoma

Follicular lymphoma

In situ follicular neoplasia

Duodenal-type follicular lymphoma

Pediatric-type follicular lymphoma

Large B-cell lymphoma with IRF4 rearrangement

Primary cutaneous follicle center lymphoma

Mantle cell lymphoma

In situ mantle cell neoplasia

Diffuse large B-cell lymphoma (DLBCL), NOS

Germinal center B-cell type

Activated B-cell type

T-cell/histiocyte-rich large B-cell lymphoma

Primary cutaneous DLBCL, leg type

EBV+ DLBCL, NOS

EBV+ mucocutaneous ulcer

DLBCL associated with chronic inflammation

Lymphomatoid granulomatosis

Primary mediastinal (thymic) large B-cell lymphoma

Intravascular large B-cell lymphoma

ALK+ large B-cell lymphoma

Plasmablastic lymphoma

Primary effusion lymphoma

HHV8+ DLBCL, NOS

Burkitt lymphoma

Burkitt-like lymphoma with 11q aberration

High-grade B-cell lymphoma, with MYC and BCL2 and/or BCL6 rearrangements

High-grade B-cell lymphoma, NOS

B-cell lymphoma, unclassifiable, with features intermediate between DLBCL and classical Hodgkin lymphoma

Mature T and NK neoplasms

T-cell prolymphocytic leukemia

Aggressive NK-cell leukemia

Adult T-cell leukemia/lymphoma

Extranodal NK-/T-cell lymphoma, nasal type

Enteropathy-associated T-cell lymphoma

Monomorphic epitheliotropic intestinal T-cell lymphoma

Indolent T-cell lymphoproliferative disorder of the GI tract

Hepatosplenic T-cell lymphoma

Subcutaneous panniculitis-like T-cell lymphoma

Peripheral T-cell lymphoma, NOS

Angioimmunoblastic T-cell lymphoma

Follicular T-cell lymphoma

Nodal peripheral T-cell lymphoma with TFH phenotype

Anaplastic large-cell lymphoma, ALK

Anaplastic large-cell lymphoma, ALK−

Breast implant–associated anaplastic large-cell lymphoma

Hodgkin lymphoma

Nodular lymphocyte predominant Hodgkin lymphoma

Classical Hodgkin lymphoma

Nodular sclerosis classical Hodgkin lymphoma

Lymphocyte-rich classical Hodgkin lymphoma

Mixed cellularity classical Hodgkin lymphoma

Lymphocyte-depleted classical Hodgkin lymphoma

***Lymphoma subtypes excluded of the study:***

Chronic lymphocytic leukemia/small lymphocytic lymphoma

Hairy cell leukemia and variant

Lymphoplasmacytic lymphoma

Waldenström macroglobulinemia

Primary DLBCL of the central nervous system

T-cell large granular lymphocytic leukemia

Chronic lymphoproliferative disorder of NK cells

Mycosis fungoides

Sézary syndrome

Primary cutaneous T cell lymphomas

Post-transplant lymphoproliferative disorders
