# Supplementary material for: A French multicentric prospective prognostic cohort with epidemiological, clinical, biological and treatment information to improve knowledge on lymphoma patients: study protocol of the “REal world dAta in LYmphoma and survival in adults” (REALYSA) cohort
Source: BMC Public Health. 2021 Mar 2;21:432. doi: 10.1186/s12889-021-10433-4 (PMC7927409; doi:10.1186/s12889-021-10433-4)
Supplement: Supplementary file 2 — Additional file 2. Inclusion procedure in the REALYSA study. Overview of the inclusion procedure in the REALYSA study. [file 12889_2021_10433_MOESM2_ESM.pptx]

## Slide 1
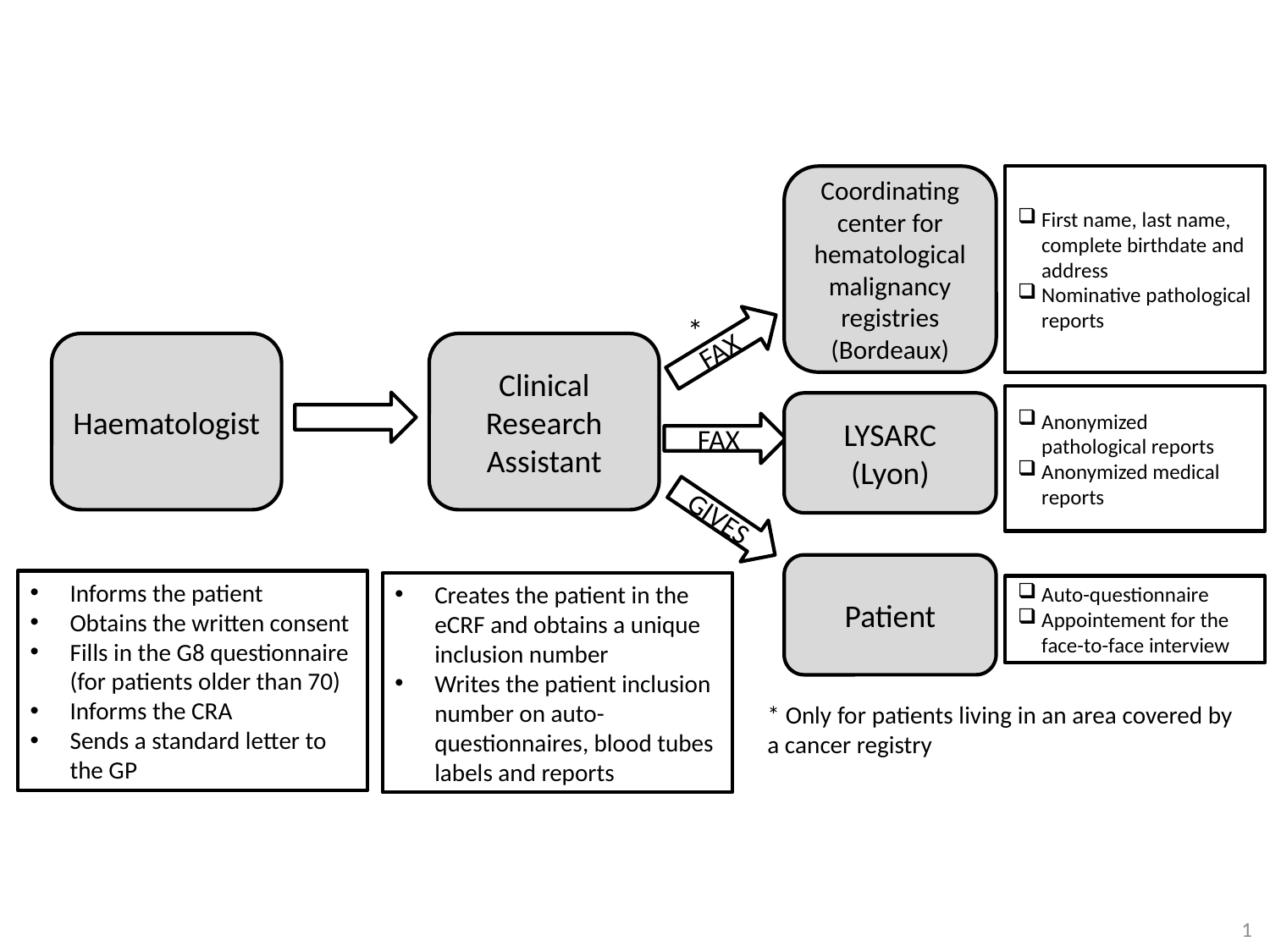

Coordinating center for hematological malignancy registries (Bordeaux)
First name, last name, complete birthdate and address
Nominative pathological reports
FAX
Clinical Research Assistant
Haematologist
Anonymized pathological reports
Anonymized medical reports
LYSARC (Lyon)
FAX
GIVES
Patient
Informs the patient
Obtains the written consent
Fills in the G8 questionnaire (for patients older than 70)
Informs the CRA
Sends a standard letter to the GP
Creates the patient in the eCRF and obtains a unique inclusion number
Writes the patient inclusion number on auto-questionnaires, blood tubes labels and reports
Auto-questionnaire
Appointement for the face-to-face interview
*
* Only for patients living in an area covered by a cancer registry
1
